# Protection with a collagen wound matrix containing polyhexamethylene biguanide supports innate wound healing in biofilm‐infected porcine wounds

**DOI:** 10.1111/wrr.70025

**Published:** 2025-04-19

**Authors:** Stephen C. Davis, Justin T. Avery, Joel Gil, Michael R. Solis, Ivan Jozic, Kelly A. Kimmerling, Katie C. Mowry

**Affiliations:** ^1^ Dr. Phillip Frost Department of Dermatology and Cutaneous Surgery, Miller School of Medicine University of Miami Florida USA; ^2^ Research & Development Organogenesis Discovery Center Birmingham Alabama USA

**Keywords:** antimicrobial barrier, biofilm, collagen dressing, MRSA, polyhexamethylene biguanide, wound healing

## Abstract

Over 90% of chronic wounds have biofilm infections, making the need for inhibiting reformation of biofilm post‐debridement paramount to support progression through the normal phases of wound healing. Herein, we describe a porcine wound model infected with methicillin‐resistant *Staphylococcus aureus* (MRSA) and examine the ability of an antimicrobial barrier composed of native type I collagen and polyhexamethylene biguanide (PCMP) to serve as a barrier to protect wounds and support progression through the innate wound healing cascade. Wounds were inoculated with MRSA and allowed to form a biofilm for 72 h, subjected to standard of care sharp debridement, then either left untreated or received PCMP for 5, 10, 15 or 20 days. Wounds were assessed for bioburden, wound closure and expression of genes related to wound healing. Wounds treated with PCMP exhibited statistically lower MRSA levels compared to untreated controls and achieved 90% closure by 2 weeks of treatment. Gene expression analysis demonstrated that by reducing bacterial load, wounds progressed through the innate wound healing cascade, while untreated wounds exhibited a dampening of the immune response. Additionally, for randomly assigned wounds, PCMP was not reapplied at dressing changes to assess the impact of inconsistent wound protection. At all timepoints, a resurgence in bioburden was observed following removal of PCMP if the wounds had not fully closed. This study highlights the value of PCMP as an antimicrobial barrier and the importance of protecting wounds through closure and resolution.

**Abbreviations**
AAALAC: American Association for Accreditation of Laboratory Animal Care
CCL2: C‐C motif ligand 2/monocyte chemotactic protein 1 (MCP‐1)
CD40L: cluster of differentiation 40 Ligand
CFU: colony forming unit
ECM: extracellular matrix
EPS: extracellular polymeric substance
GO: gene ontology
IL‐: interleukin
MCL: Markov Clustering Algorithm
MMP: matrix metalloproteinase
MRSA: methicillin resistant *Staphylococcus aureus*
PCMP: Porcine collagen matrix with PHMB
PHMB: polyhexamethylene biguanide
REVIGO: reduce and visualise gene ontology
STRING: search tool for the retrieval of interacting genes/proteins
TNF: tumour necrosis factor

## 1 INTRODUCTION

In 2017, biofilms were estimated to have had a global economic cost of $281 billion within wound care.^1^ Biofilms are particularly difficult to address clinically as they consist of a high concentration of bacteria (10^8^–10^11^ cells/g) alongside an extracellular polymeric substance (EPS) that protects the bacteria by allowing them to adhere to one another, aggregate and promote cell–cell communication.^2^ With estimations of biofilms being present in 78% to over 90% of chronic wounds,^3^, ^4^ the ability to combat biofilm is paramount to promoting wound healing. While preparation of the wound bed with sharp debridement is critically important to disrupt the EPS and remove bacteria from the wound bed, it has been shown that sharp debridement alone is not sufficient, as biofilm can reform in 24–72 h post‐debridement.^5^ To protect the wound from additional insult, it is important to utilise a multimodal approach involving debridement alongside antimicrobials to reduce biofilm burden and promote healing.^6^

Several wound dressings and cleansers have been developed for wound management and function as either an antimicrobial barrier to the wound or as a wash for the wound bed with various compounds including honey, ionic silver or polyhexamethylene biguanide (PHMB).^7^ Efficacy of wound cleansers is largely dependent on the irrigant used. Tap water and saline have been shown to be ineffective at reducing bioburden, while some disinfectants can reduce bioburden but result in varying levels of cytotoxicity to various cell types involved in physiological wound healing.^8^, ^9^ Modern wound dressings include classes such as semi‐permeable films, hydrogels and bioactive dressings, which, depending on their composition and design, may promote an exchange of gases and fluid or provide a barrier to prevent additional bacteria from infecting the wound bed.^10^ Wound dressings designed with a chemical antimicrobial can protect from microbial contamination or biofouling of the dressing.^11^

Preclinical and in vitro assessments of a porcine native type 1 collagen matrix with PHMB (PCMP) demonstrated that it was effective at controlling bioburden and acting as an antimicrobial barrier.^5^ The native collagen comprising PCMP has been shown to reduce aberrant proteolytic activity traditionally found in chronic wounds and to be resistant to degradation in an in vitro chronic wound model.^12^ Furthermore, a recent case study of five patients highlighted how targeted debridement of biofilm followed by an application of PCMP was beneficial in supporting wound closure. Wounds were imaged with a fluorescence imaging camera system to determine the presence and location of bacterial loads ≥10^4^ colony forming unit (CFU)/g, debrided, after which PCMP was applied, resulting in either complete wound closure or nearly complete wound closure of chronic, non‐healing lower extremity wounds after just 6 weeks.^13^

To further evaluate how PCMP can be used as a barrier to prevent biofilm reformation, thereby supporting innate wound resolution, we utilised a porcine biofilm wound model and assessed bioburden levels, wound closure and genomic regulation of wound healing‐related genes for up to 20 days of treatment.

## 2 METHODS

### 2.1 Animal care and wound model

Female specific pathogen free (*N* = 4, SPF: Looper Farms, North Carolina) pigs weighing 35–40 kg were housed for at least 5 days prior to the initiation of experiments. Animals were fed a basal diet ad libitum and housed individually in the University of Miami's American Association for Accreditation of Laboratory Animal Care (AAALAC)‐accredited facility. Prior to surgery up until the end of the study, animals were provided analgesics for pain management. Animals were anaesthetised and large reticular dermal wounds measuring 4 cm × 4 cm and 3 mm deep were generated on the paravertebral and thoracic area with a specialised electrokeratome to ensure consistent wounds. Wounds were separated by 5–7 cm of unwounded skin to minimise the impact of cross‐contamination as well as crosstalk between wounds.

### 2.2 Wound inoculation

A pathogenic strain of methicillin‐resistant *Staphylococcus aureus* (MRSA, Strain NRS384, USA300) was used for this study. A 10^10^ CFU/mL solution in sterile water was diluted to 10^4^ CFU/mL in Tryptic Soy Broth. Immediately post wounding, 100 μL this inoculum was added into the centre of each wound site and evenly spread over the surface of the wound with a sterile spatula. All wounds were then covered in a polyurethane film dressing (Tegaderm Transparent Dressing; 3M Health Care, St. Paul, MN) for 72 h to allow for biofilm formation.^14^ Dressings were secured with surgical tape and wrapped with Coban elastic wrap (3M, St. Paul, MN).

### 2.3 Experimental product application

Purified native type 1 collagen extracellular matrix (ECM) plus PCMP (PuraPly®AM, Organogenesis, Canton, MA) is a purified porcine small intestinal submucosa‐derived native, crosslinked type I collagen matrix with PHMB.

**FIGURE 1:**
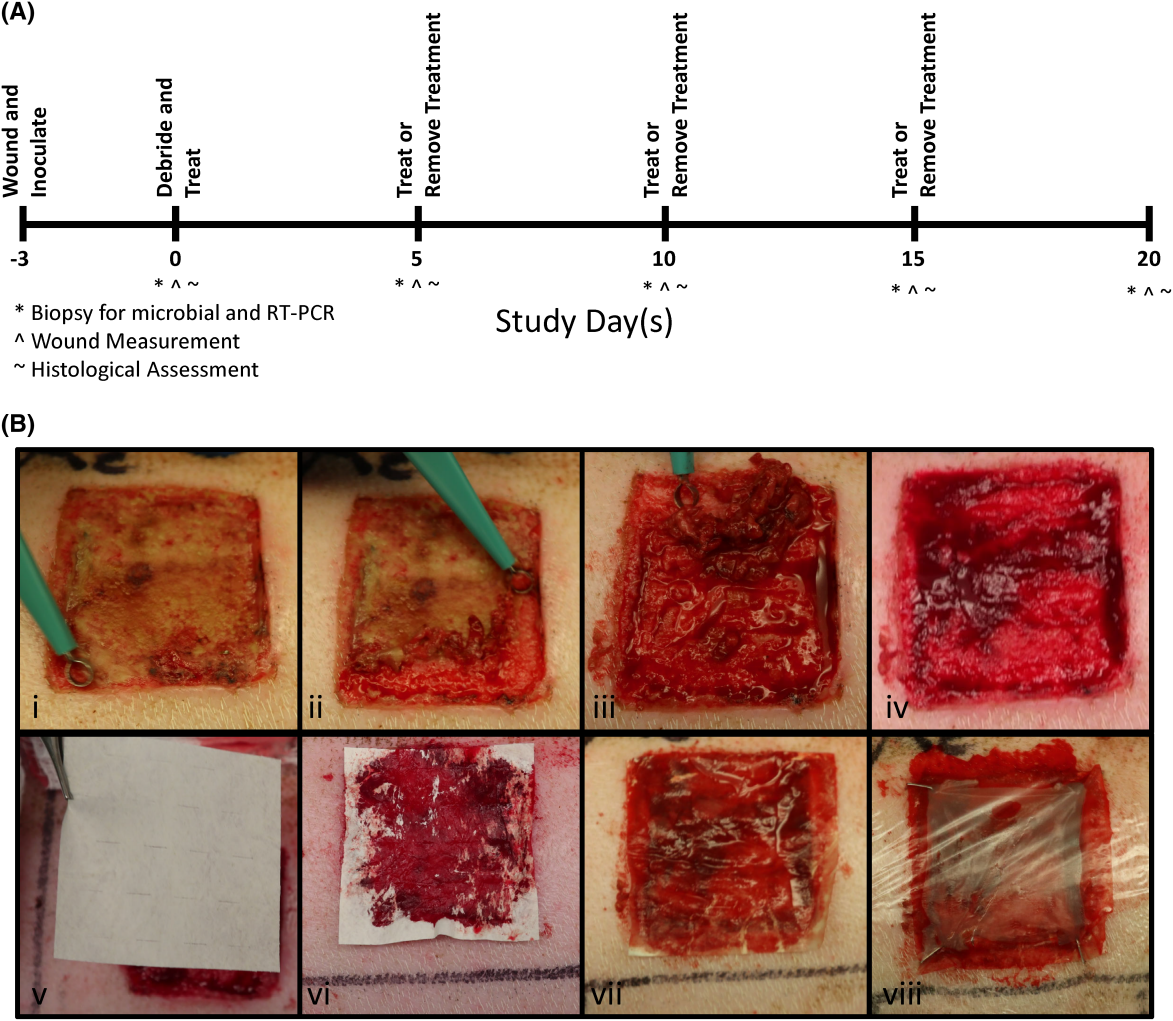
Experimental design and product application. (A) Study design timeline. (B) Representative images depicting product application. Wounds were sharply debrided (i–iv), Porcine collagen matrix with polyhexamethylene biguanide was applied to the fresh wound bed (v and vi), wetted with saline to ensure optimal contact with the wound bed (vii), then stapled and covered with secondary dressings (viii). RT‐PCR, reverse transcription‐polymerase chain reaction.

After biofilm was established, wounds underwent sharp debridement to model the standard of care using a 4 mm curette (Figure 1B, i–iv). For wounds designated as baseline wounds (two per animal), four 6 mm biopsy punches were then taken post‐debridement for bacterial quantification, and an incisional biopsy of 45 mm × 2 mm was taken across the centre of the wound for histological assessment. Wounds were randomly assigned to groups: either (1) maintaining PCMP throughout the study, (2) removal of PCMP at specified intervals throughout the study or (3) untreated controls. For wounds receiving PCMP application, 200 μL of sterile saline was used to moisten the device to ensure optimal contact with the wound bed (Figure 1B, v–vii). PCMP was secured with staples prior to covering with Tegaderm and Coban dressing (Figure 1B, viii). Wound dressings were removed every 5 days for wound assessment and bioburden quantification, at which point PCMP was reapplied or removed and covered with secondary dressings (where applicable) for up to 20 days (Figure 1A).

### 2.4 Wound closure assessment

Images were taken and wound tracings were generated at all dressing changes. Tracings and a ruler for reference were scanned and imported into ImageJ Version 1.53k (National Institute of Health, Bethesda, MD). Borders of the wounds were traced in ImageJ and used to calculate the overall area of wounds at each time point. Wound closure was calculated as a percentage of area closure compared to initial measurements for each individual wound. To interpolate theoretical wound healing rates, wound closure data was modelled as previously described.^15^ Briefly, an Exponential Plateau fit was calculated in GraphPad Prism Version 10.0.0 (GraphPad Software, Boston, MA) and values were plugged into the following equation to determine the predicted day at which various percent closures would be achieved:

$$Y_{\mathrm{Max}}-\left(Y_{\mathrm{Max}}-Y_{0}\right)\times e^{-k\times \text{days after treatment}}.$$

### 2.5 Histological assessment

Incisional biopsies across the centre of the wound and extending into the normal adjacent skin were taken (45 mm × 2 mm). Biopsies were split in half due to size constraints of the processing equipment, with half of each wound observed per slide from normal adjacent skin to the centre of the wound bed. Biopsies were processed, sectioned and stained using standard histological methods. Hematoxylin and Eosin (H&E)‐stained slides were blinded and analysed for five elements: (1) *percentage of wound reepithelialization*—measurement of length of the wound surface that had been covered with epithelium; (2) *epithelial thickness*—five equal distanced points were measured and averaged to determine the mean epithelial thickness; (3) *white cell infiltrate*—qualitative assessment based on the presence and amount of subepithelial mixed leukocyte infiltrates with a 1 = absent, 2 = mild, 3 = moderate, 4 = marked, 5 = exuberant; (4) *Granulation tissue formation*—qualitative assessment based on the percentage of granulation tissue formed where 1 = <5%, 2 = 6%–25%, 3 = 26%–50%, 4 = 51%–75%, 5 = 76%–100% and (5) *New blood vessel formation*—qualitative assessment where the presence of new blood vessels was scored where 1 = absent, 2 = mild, 3 = moderate, 4 = marked and 5 = exuberant.

### 2.6 Reverse transcription‐polymerase chain reaction assessment

Two 4 mm biopsy punches were taken at 0, 5, 10, 15 and 20 days as well as four 4 mm biopsy punches from unwounded skin for comparison. Biopsies were stored in RNA*later* (Invitrogen, Carlsbad, CA) and extraction of RNA was done using the RNeasy Fibrous Tissue Mini Kit (Qiagen, Germantown, MD). RNA was quantified and complementary DNA (cDNA) was generated with the RT^2^ First Strand cDNA kit (Qiagen, Germantown, MD) using 500 ng RNA. RT^2^ Pig Wound Healing Profiler Arrays (Qiagen, Germantown, MD) were used to assess over 80 genes related to normal wound healing. Fold change was calculated as 2^−ΔΔCt^ and graphed as a Log2 transformation.^16^ Hierarchical cluster analysis was performed using JMP version 18.0.1 (SAS Institute Inc., Cary, NC) using Ward methodology.^17^

### 2.7 Search Tool for the Retrieval of Interacting Genes and reduce and visualise gene ontology analysis

Log2‐transformed fold change values were used to determine genes that were statistically significant by comparing timepoints to unwounded skin. Statistically significant genes were input into string-db.org Search Tool for the Retrieval of Interacting Genes/Proteins (STRING, version 12).^18^ Genes were clustered using a Markov Cluster Algorithm (MCL) inflation rate of 3, and gene ontology (GO) terms for each cluster were assessed. GO terms and their false discovery rate were put into reduce and visualise gene ontology (REVIGO) to pare down the list of relevant GO terms.^19^

### 2.8 Statistical analysis

All statistics were performed using GraphPad Prism version 10.0.0 (GraphPad Software, Boston, MA). For theoretical closure modelling, an exponential plateau fit was performed. For fold reduction of bioburden, Brown‐Forsythe and Welch analysis of variance (ANOVA) with Dunnett's T3 multiple comparisons test was performed. For string analysis, Welch's *T*‐test with Holm‐Šídák multiple comparisons was performed. For wound closure comparisons of treatment versus removal of treatment, Welch's *T*‐test was performed.

## 3 RESULTS

### 3.1 Debridement and treatment with PCMP reduce bioburden and support wound closure

**FIGURE 2:**
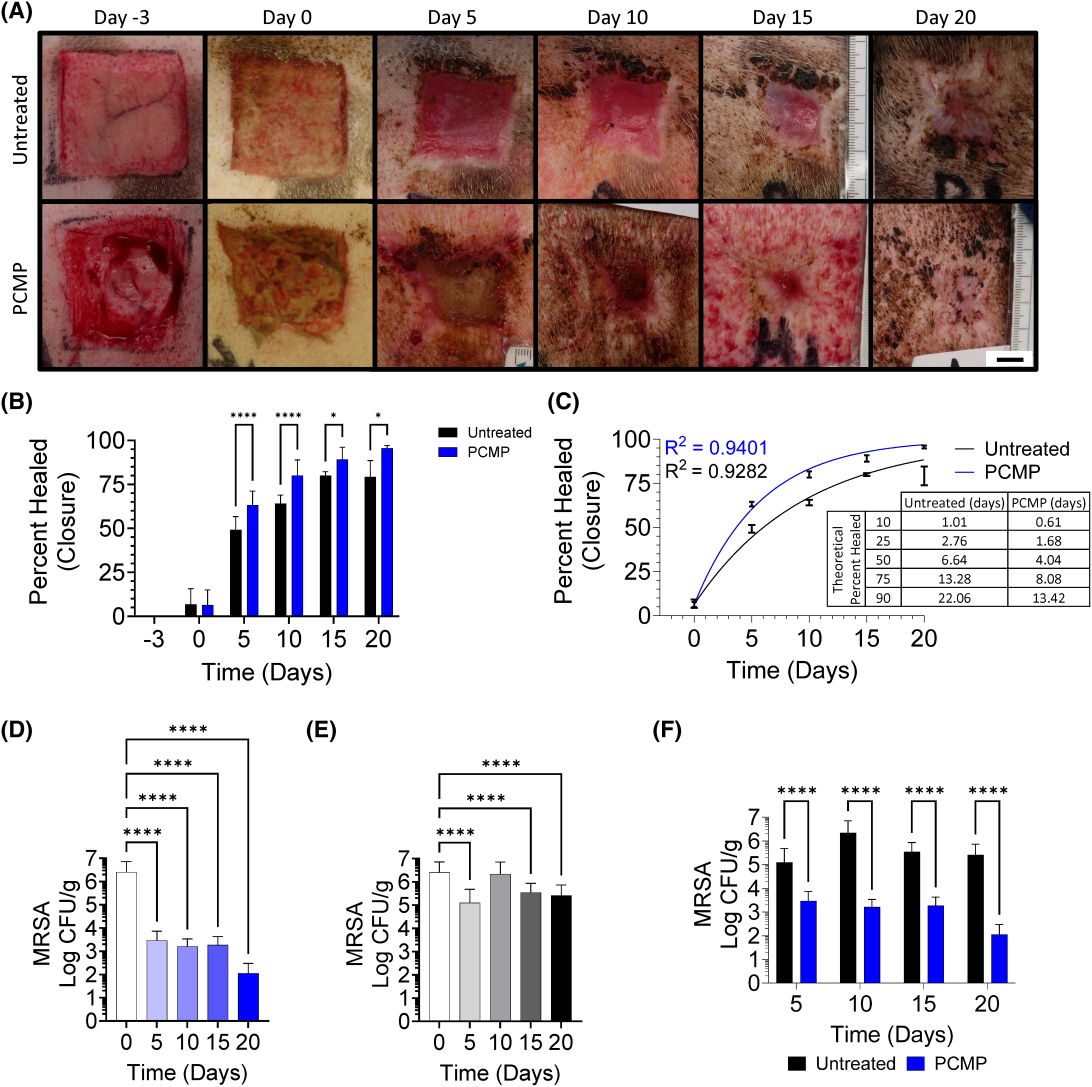
Ongoing coverage with Porcine collagen matrix with polyhexamethylene biguanide (PCMP) resulted in wound closure by reducing bioburden. (A) Representative images of wounds at different stages of assessment for PCMP and untreated wounds. Scale bar set to 1 cm. (B) Wound closure calculated as percent area normalised to initial wound size. (C) Theoretical modelling of wound healing determined PCMP‐treated wounds would reach 50% closure during first application and 90% closure by 2 weeks whereas untreated wounds would take up to a week to reach 50% closure and 3 weeks to achieve 90% closure. (D) PCMP treatment resulted in statistical reduction in MRSA at all timepoints compared to baseline‐debrided wounds with greater than four‐fold log reduction at the end of study. (E) Untreated controls achieved statistical decreases in methicillin‐resistant *Staphylococcus aureus* (MRSA) on Days 5/15/20, but never had greater than a two‐fold log reduction. (F) PCMP‐treated wounds had statistically less MRSA when compared to untreated controls. Average ± standard deviation reported; *p ≤ 0.05; ****p < 0.0001.

Large wounds were generated, inoculated with 10^4^ CFU/mL MRSA, and left for 72 h to allow biofilm to develop and mature. Wounds were then sharply debrided to remove the biofilm and either left untreated or treated with PCMP for up to 20 days before coverage with Tegaderm and Coban (Figure 1). As expected, debridement and application of PCMP resulted in statistically increased closure compared to untreated controls (Figure 2A,B). An exponential plateau fit was performed on wound closure measurements to determine predicted or theoretical wound closure, mirroring the experimental findings, where 90% wound closure was observed in less than 2 weeks for wounds treated with PCMP compared to 75% wound closure for untreated controls at that same timepoint (Figure 2C). PCMP application following sharp debridement resulted in statistically decreased bioburden at all timepoints, with a >4 log fold reduction by the end of study (Figure 2D). Untreated controls, while having statistically decreased levels on Days 5, 15 and 20, only had at maximum a single log fold reduction in MRSA, which is statistically significant but not biologically relevant (Figure 2E). Comparing untreated controls to PCMP‐treated wounds, a statistically significant decrease in overall bioburden was observed at all timepoints (Figure 2F).

### 3.2 Assessment of wound resolution by RT‐PCR

**FIGURE 3:**
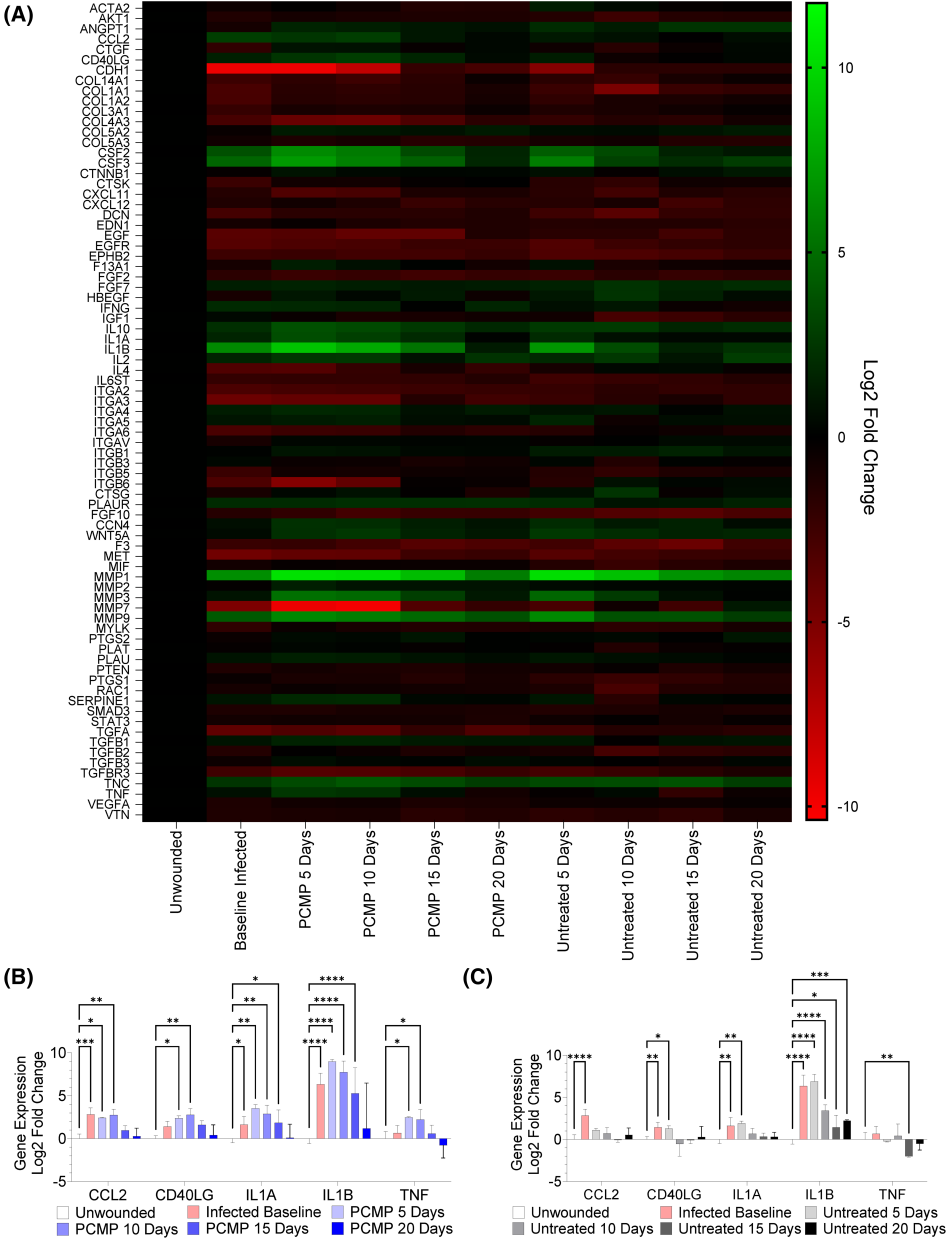
Wound healing gene expression markers with ongoing Porcine collagen matrix with polyhexamethylene biguanide (PCMP) coverage. (A) ΔΔCt analysis performed using unwounded skin as baseline expression. Heatmap expressed as Log2 fold change in expression. (B and C) Inflammatory markers for wound treated with PCMP or untreated, respectively. Chemoattractant (C‐C motif ligand 2/monocyte chemotactic protein 1 (MCP‐1) [CCL2]), activation markers (cluster of differentiation 40 Ligand [CD40LG]) and inflammation molecules (IL1A, IL1B and tumour necrosis factor [TNF]) are differentially regulated depending on the progression of the wound and product application. Average ± standard deviation reported; *p ≤ 0.05, **p ≤ 0.01, ***p ≤ 0.001, ****p < 0.0001. IL‐, interleukin.

**FIGURE 4:**
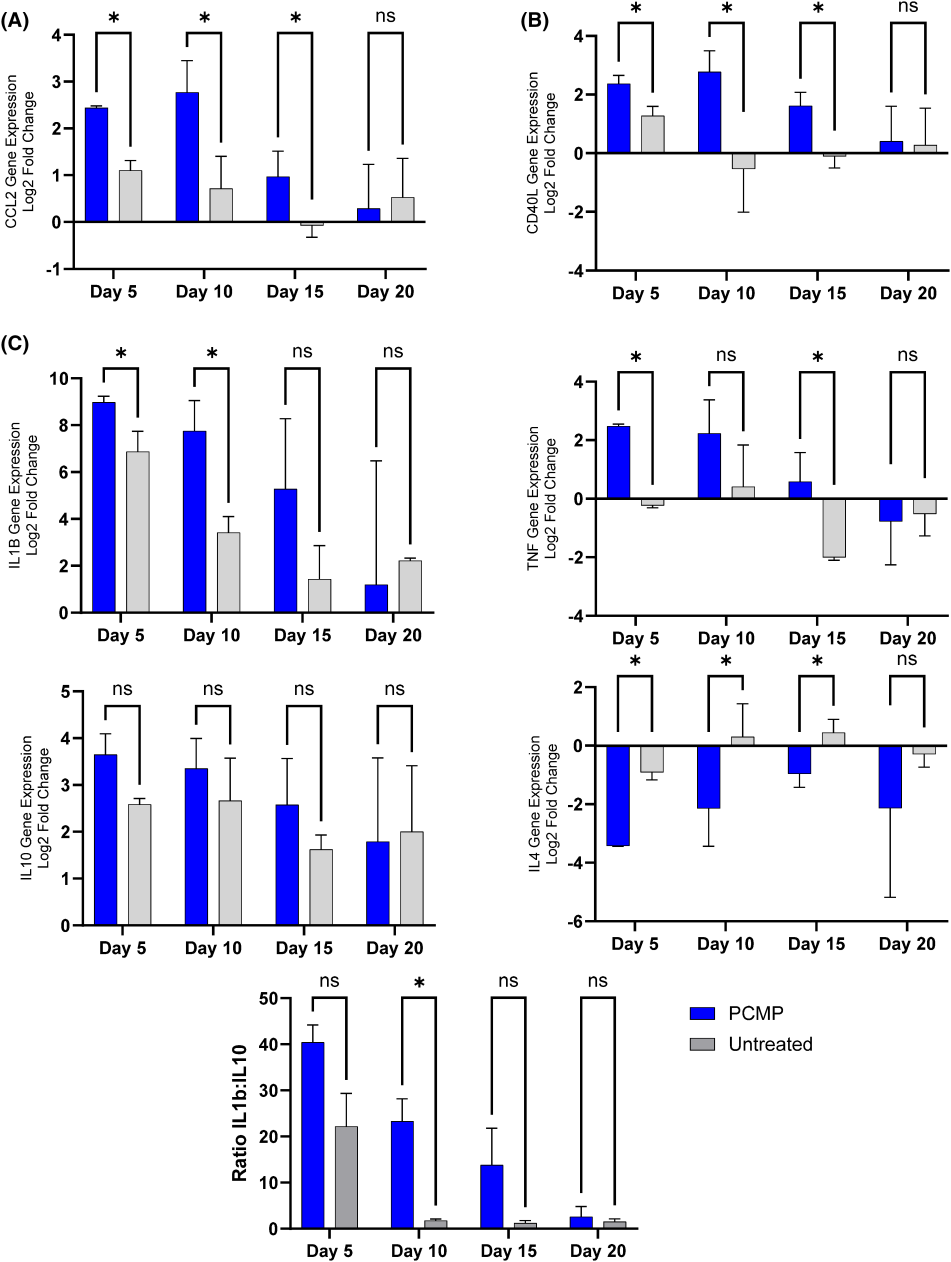
Application of Porcine collagen matrix with polyhexamethylene biguanide (PCMP) supports clearance of methicillin‐resistant *Staphylococcus aureus* by preventing biofilm reformation and allowing inflammation to persist. (A) Wounds treated with PCMP resulted in statistically elevated recruitment of monocyte/macrophages through C‐C motif ligand 2/monocyte chemotactic protein 1 (MCP‐1) (CCL2) and (B) subsequent activation with cluster of differentiation 40 Ligand (CD40L) compared to untreated control. (C) Statistically elevated effector inflammatory response (IL‐1β and tumour necrosis factor [TNF]) and a ratio of IL‐1β:IL‐10 indicating a strong M1 like phenotype for PCMP‐treated wounds compared to untreated wounds. Average ± standard deviation reported; *p ≤ 0.05, IL‐, interleukin; ns, not significant.

Biopsies taken from wounds were analysed using a porcine wound healing array to evaluate progression through the stages of wound healing to closure (Figure 3A). While several pathways of interest were identified, the most interesting involved the recruitment, activation and activity of monocyte/macrophages to the infected wound bed when comparing untreated and treated wounds. For PCMP‐treated wounds, macrophage response genes had time‐dependent regulation shifts over the course of the study, with (1) chemokine ligand 2 (CCL2) exhibiting a statistically significant increase in response to biofilm infection, (2) up‐regulation of monocyte/macrophage activation marker cluster of differentiation 40 ligand (CD40LG) as cells were recruited to the site of insult and (3) elevated inflammatory response cytokines interleukin (IL)‐1α, IL‐1β and tumour necrosis factor (TNF) during the first 10 days, which later resolved by Day 20 (Figure 3B). For untreated controls, the initial response after infection was similar, with a statistically significant increase in chemokines and cytokine response after infection; however, the profile did not follow the same response over time. Both recruitment and activation markers CCL2 and CD40LG lost significance by Days 5 and 10, respectively, which in turn resulted in a decreased inflammatory phenotype as observed by diminished IL‐1α, IL‐1β and TNF response (Figure 3C). When comparing the groups, PCMP‐treated wounds had a statistically significant increase in expression of recruitment and activation chemokines/cytokines as well as inflammation markers IL‐1β and TNF (Figure 4). No statistically significant difference in the anti‐inflammatory cytokine IL‐10 was observed, while IL‐4 did show a statistical difference at Days 5, 10 and 15 (Figure 4). To further assess the inflammatory process, a ratio of IL‐1β: IL‐10 fold‐change was performed, which highlighted how untreated wounds quickly lose an inflammatory M1 response by Day 10 as biofilm resurgence occurs. Conversely, PCMP‐treated wounds exhibited a steady decrease in the inflammation ratio as the bioburden was resolved and the wound progressed towards closure (Figure 4).

### 3.3 Qualitative assessment of wound resolution using gene ontology terms

**FIGURE 5:**
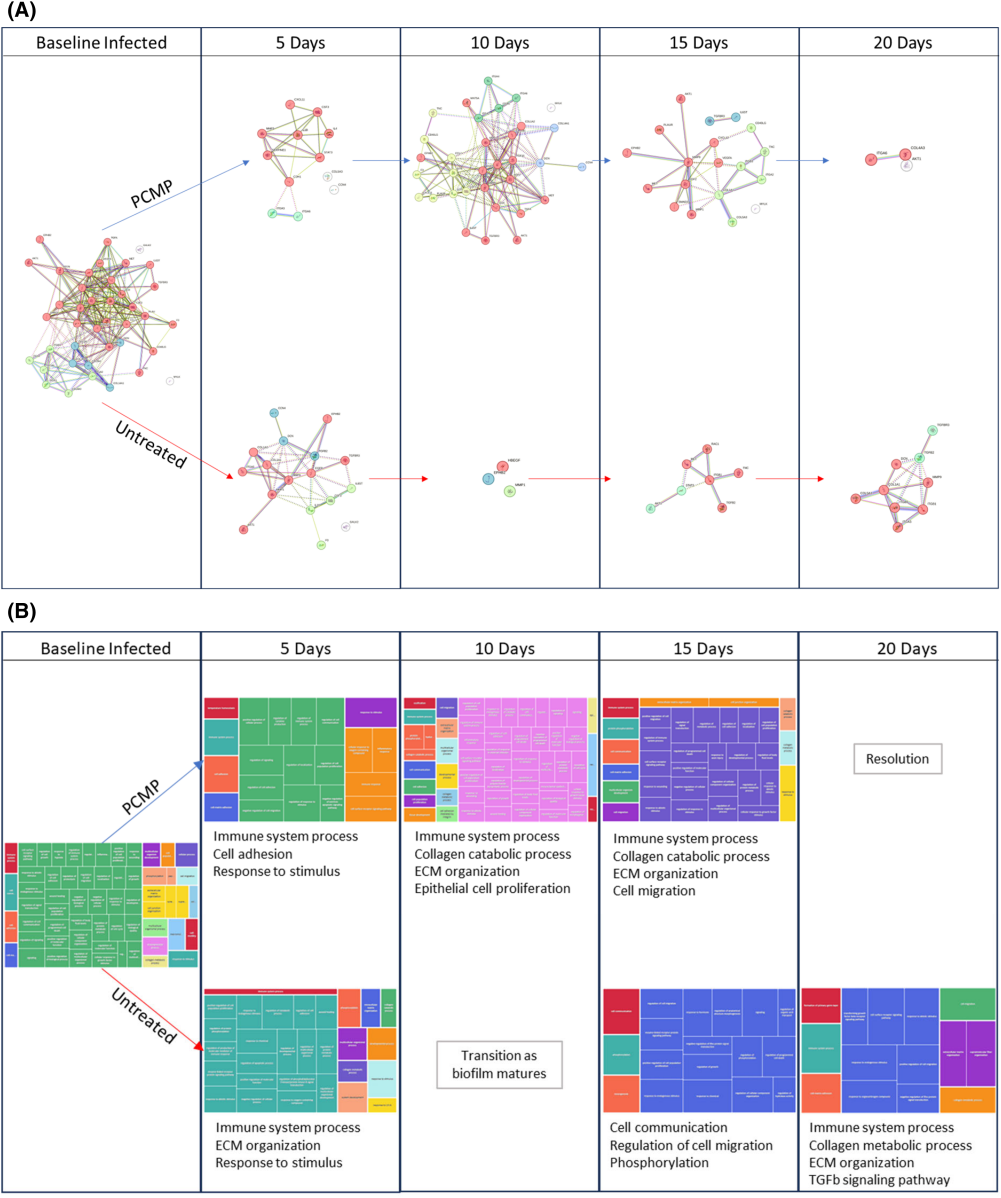
Gene ontology (GO) terms illustrate how wounds progress from inflammation to proliferation/remodelling phases of wound healing. (A) search tool for the retrieval of interacting genes/proteins assessment on statistically up/down regulated genes from Infected baseline Day 0, Porcine collagen matrix with polyhexamethylene biguanide (PCMP)/Untreated Day 5, PCMP/Untreated Day 10, PCMP/Untreated Day 15 and PCMP/Untreated Day 20. A list of statistically relevant genes and their associated gene counterparts can be found in Table S1. (B) Extracted GO terms were reduced with reduce and visualise gene ontology to remove redundant terms. Terms and their Term IDs can be found in Tables S2 and S3. ECM, extracellular matrix.

Statistically significant shifts in gene expression were assessed by STRING to determine the overarching biological processes and GO terms implicated throughout the wound healing process (Figure 5A; Table S1). To remove redundant GO terms and provide a more concise picture of the processes involved at various stages of healing, GO terms from STRING assessment were input into REVIGO. For wounds that received PCMP treatment, GO terms transitioned from being associated with the inflammatory responses to terms associated with the proliferation and remodelling phases (Figure 5B; Tables S2 and S3). After biofilm maturation, GO terms associated with cell recruitment/adhesion, inflammatory response and ECM organisation were observed. After 5 days, immune/inflammatory response, cellular proliferation and cell adhesion GO terms indicated the wound was still within the inflammatory phase. By Day 10 following treatment of wounds with PCMP, continued expression of immune response was observed, but further GO terms associated with ECM reorganisation, epithelial proliferation and response to growth factor stimulus began to emerge, indicating a shift towards the remodelling phase of wound healing. By Day 15, terms associated with epithelial migration, collagen catabolism and cell junction organisation were observed, indicating a shift towards the remodelling phase. Day 20 after wounds were treated with PCMP, no GO terms were associated with genes of statistical interest, marking a return to homeostasis.

When wounds were left untreated after debridement, wounds mirrored a similar Day 5 response as PCMP‐treated wounds, with GO terms being associated with immune system process, collagen catabolic/metabolic process and response to stimulus (Figure 5B; Tables S2 and S3). Contrary to PCMP‐treated wounds, those left untreated for 10 days exhibited no GO terms as there were little statistically up/down regulated genes, indicating a shift in profile. Wounds left untreated for 15 days exhibited GO terms associated with cell migration/communication, regulation of programmed cell death and cell population proliferation. By Day 20, GO terms associated with immune system processes were observed again, as well as anti‐inflammatory terms, including transforming growth factor beta receptor signalling pathway. When observed alongside the MRSA levels for these wounds (Figure 2D), maturation of the biofilm could be associated with the shift in regulation as the biofilm promotes an anti‐inflammatory response.^20^

### 3.4 Treatment of wounds with PCMP through closure protected wounds from biofilm reformation

**FIGURE 6:**
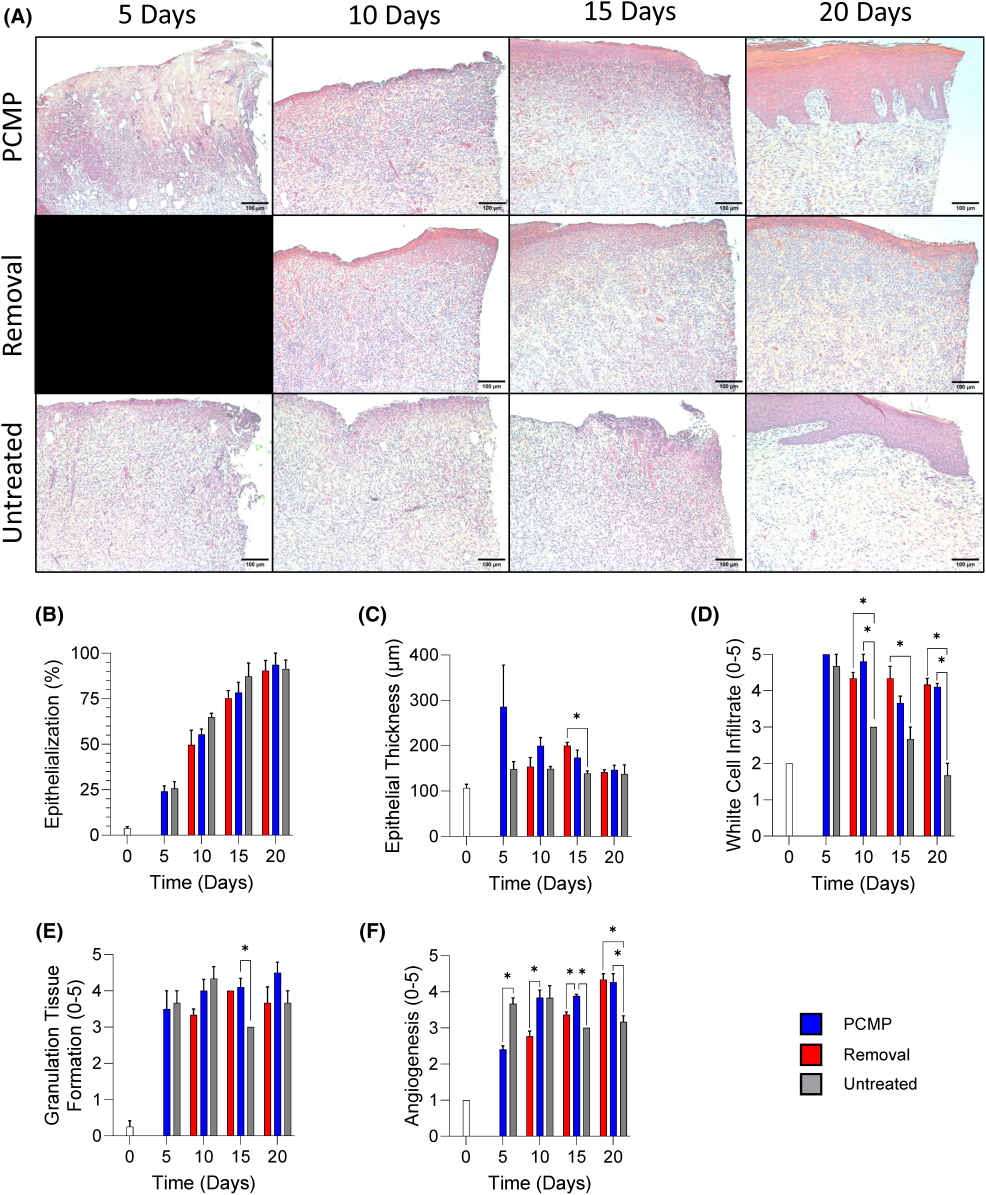
Histological assessment. (A) Representative hematoxylin and eosin images from the centre of the wound bed. Images captured at 10×. Scale bar set to 750 μm. (B–F) Histological scoring for percent epithelialization, epithelial thickness, white cell infiltrate, granulation tissue formation and angiogenesis. Details on scoring can be found in the methods. Multiple unpaired *T*‐tests, Holm‐Šídák multiple comparisons. Average ± standard deviation reported; *p < 0.05. PCMP, Porcine collagen matrix with polyhexamethylene biguanide.

**FIGURE 7:**
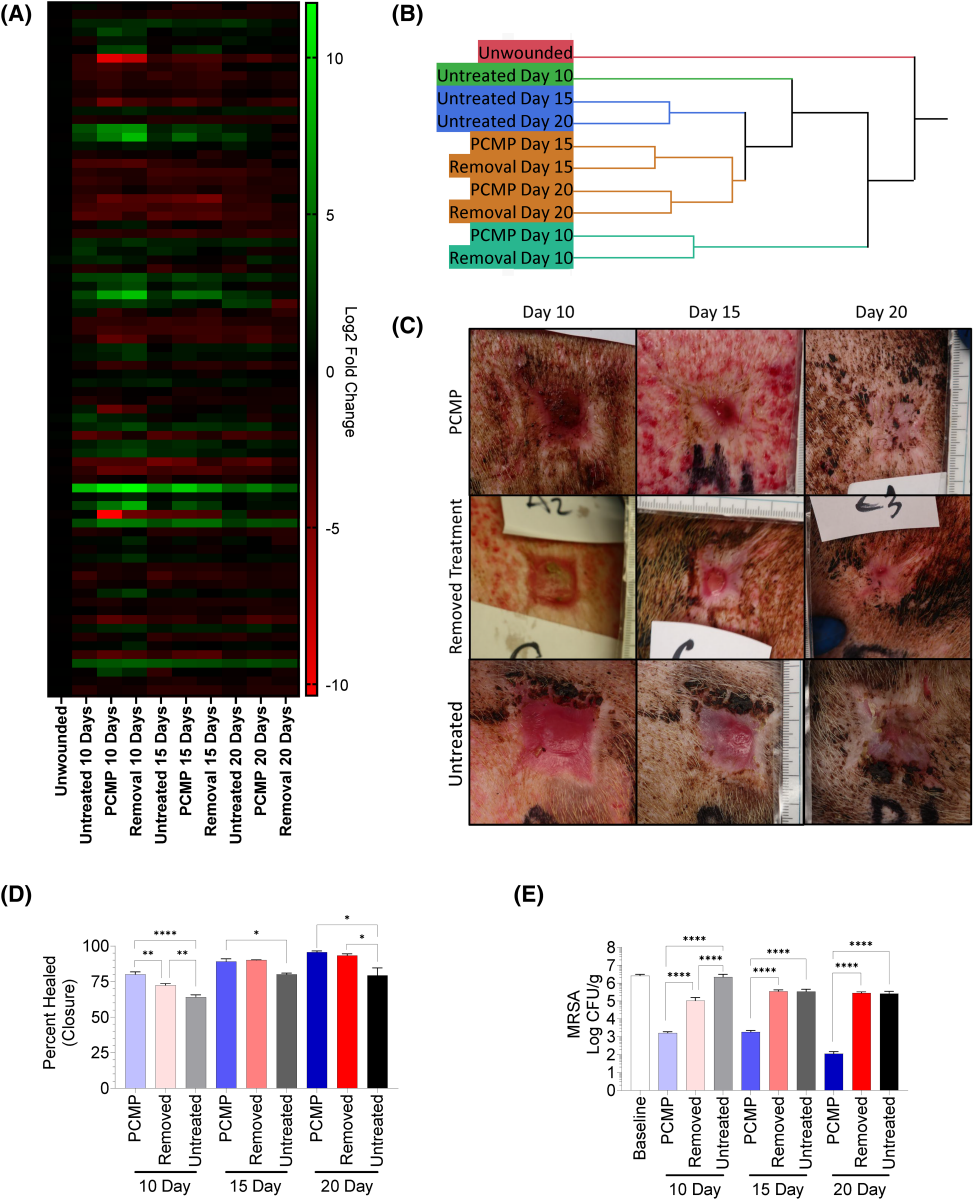
Removal of Porcine collagen matrix with polyhexamethylene biguanide (PCMP) barrier prior to closure allows for biofilm reformation. (A) ΔΔCt analysis performed using unwounded skin as baseline expression. Heatmap expressed as Log2 fold change in expression. (B) Hierarchical cluster analysis dendrogram. (C) Representative images of wounds on Days 10, 15 and 20. (D) Percent wound closure assessment. (E) Bioburden assessment where regardless of timepoint, barrier removal allowed for a statistical resurgence of bioburden compared to continued treatment with PCMP. One‐way ANOVA (Tukey or Brown‐Forsythe and Welch multiple comparisons depending on whether standard deviations were statistically different by Bartlett's test). *p ≤ 0.05, **p ≤ 0.01, ****p < 0.0001.

Treatments were removed every 5 days to allow for wound assessments. For randomly assigned wounds, PCMP was not reapplied at either Days 5, 10 or 15 to determine the impact of PCMP removal and the value of utilising an antimicrobial barrier through various stages of wound healing (Figure 1A). Representative images taken from the centre of the wound bed of untreated, PCMP‐treated and removal of treatment wounds show high levels of inflammation and immune cell infiltration (Figure 6A). A series of histological assessments including epithelialization, epithelial thickness, white cell infiltrate, granulation tissue formation and angiogenesis was performed (Figure 6B–F). White cell infiltrate levels for untreated matched the expression assessment found in Figure 4, with lower qualitative scoring for untreated wounds. Wounds were further assessed by reverse transcription‐polymerase chain reaction (RT‐PCR) assessment (Figure 7A) and hierarchical cluster analysis was performed, resulting in PCMP‐treated wounds and product removed clustering together, while untreated wounds clustered separately (Figure 7B). When assessing wound closure, all treatment methodologies were statistically different from one another on Day 10, with PCMP‐treated wounds having the greatest percent closure (Figure 7C,D). Interestingly, quantitative microbial assessment demonstrated increased resurgence of MRSA levels detected at all time points when wounds were left untreated or if PCMP was removed for up to 5 days (Figure 7E).

## 4 DISCUSSION

In this porcine biofilm model, sharp debridement and protection of wounds with an antimicrobial barrier that reduced bacterial burden was utilised. Assessments of wound healing‐related gene expression found transitions in gene profiles associated with normal progression through the wound healing process following treatment of wounds with PCMP throughout healing to closure, including inflammation at early timepoints and remodelling by later timepoints. Untreated wounds, on the other hand, exhibited a shift in gene profile as planktonic bacteria regenerated the biofilm and resulted in an anti‐inflammatory phenotype in the monocyte/macrophages to prevent clearance of the bacteria/biofilm. Additionally, removal of PCMP resulted in reformation of biofilm, resulting in a significant impact on percent closure at early timepoints. These results highlight the importance of protecting the wound bed from biofilm reformation using an antimicrobial barrier.

Sharp debridement has long been the gold standard for treatment of biofilm, promoting both the removal of necrotic tissue and infected tissue.^21^ It disrupts the biofilm by removing the EPS and putting bacteria into a planktonic state; however, biofilms can quickly be reformed if left unchecked.^22^ It is well established that debridement alone is not sufficient to disrupt and resolve biofilm completely.^23^ By applying PCMP to debrided wounds, we observed a 3–4 log fold reduction in bioburden throughout the course of the study without observing an increase in CFU at any point after debridement.

Biofilms have been shown to impede wound healing through several mechanisms, such as modulation of the immune microenvironment and enhancing a proteolytic environment with elevated levels of MMPs.^24^, ^25^, ^26^ Resolution of abnormal levels of proteolytic enzymes is a critical part of wound management to support allowing wounds to progress towards closure.^27^ Native collagen ECMs have been shown to reduce the activity of a wide range of MMPs including collagenases, gelatinases and stromelysins.^12^, ^28^ By combining a native porcine collagen product with PHMB, PCMP not only acts as a barrier to bacteria and recolonization but dampens the proteolytic environment by providing a sacrificial target for aberrant MMP expression.

Wounds were observed every 5 days to allow for assessments of clinical wound closure and microenvironmental changes. Since PCMP reduced levels of MRSA found within the wound bed, we observed an efficient transition from the acute early inflammatory state and progression through the innate wound healing cascade. This finding is key, given the propensity for chronic wounds to stall in the inflammatory phase.^4^ While biofilms are classically thought to result in persistent levels of inflammation, it is also important to remember that as biofilm matures, it will alter the immune microenvironment to an anti‐inflammatory phenotype, especially in macrophages, to prevent macrophage recruitment and phagocytosis.^20^ For untreated wounds, we observed this phenotype, with GO and RT‐PCR assessment highlighting an early shift in the microenvironment of these wounds towards an anti‐inflammatory environment with decreased CCL2, CD40L and inflammatory markers like IL‐1β as quickly as 5 days post‐debridement.

One of the limitations of this study is the lack of chronicity in this model. While porcine models are well documented as an excellent tool for modelling human wound healing,^29^ the lack of long‐term chronicity prevents it from fully representing the complicated wound microenvironment of chronic wounds like diabetic foot ulcers (DFUs), which are clinically related to a number of other complications not represented in standard animal models. However, these findings do mirror the clinical observations previously published with this product.^13^ Additionally, this study modelled biofilms with a MRSA300, a community‐associated MRSA that has developed a multidrug‐resistance over the last several years.^30^ While studying the effects of an antimicrobial like PHMB on this strain alone is important, clinical biofilms often consist of multiple bacterial strains,^31^ suggesting the applicability of additional studies addressing polymicrobial biofilms.

In conclusion, this study highlighted how PCMP is an effective antimicrobial barrier, while the removal of PCMP treatment prior to wound closure resulted in a resurgence of bioburden. By preventing biofilm reformation with PCMP, wounds were able to efficiently progress from the inflammatory phase to the remodelling phase through the innate wound healing cascade.

## AUTHOR CONTRIBUTIONS

**SCD**: study design, analysis and interpretation of data, draft of manuscript. **JTA**: study design and data collection, analysis and interpretation of data, draft of manuscript. **JG**: data collection, analysis of data, manuscript revision. **MRS**: data collection, analysis of data, manuscript revision. **IJ**: study design, data collection, analysis and interpretation of data, manuscript revision. **KAK**: study design, interpretation of data, manuscript revisions. **KCM**: study design, interpretation of data, manuscript revisions. All authors approved the final manuscript.

## CONFLICT OF INTEREST STATEMENT

No conflicts of interest for Stephen Davis, Joel Gil, Michael Solis and Ivan Jozic. Justin Avery, Kelly Kimmerling and Katie Mowry are employees of Organogenesis.

## Supporting information


**Table S1.** STRING assessment genes. Statistically up/down regulated genes were imported into string-db.org and clustered using MCL clustering (inflation parameter 3).

**Table S2.** REVIGO terms for PCMP wounds. (A) Unwounded versus Infected Day 0; (B) Unwounded versus Day 5 Treated; (C) Unwounded versus Day 10 Treated; (D) Unwounded versus Day 15 Treated. Colour correlates with clustered terms per REVIGO assessment.

**Table S3.** REVIGO terms for untreated wounds. (A) Unwounded versus Infected Day 0; (B) Unwounded versus Day 5 Untreated; (C) Unwounded versus Day 15 Untreated; (D) Unwounded versus Day 20 Untreated. Colour correlates with clustered terms per REVIGO assessment.

## Data Availability

Supporting data are available upon reasonable request from the corresponding author.
