# Supplementary figures and images for: Protection with a collagen wound matrix containing polyhexamethylene biguanide supports innate wound healing in biofilm‐infected porcine wounds
Source: Wound Repair Regen. 2025 Apr 19;33(2):e70025. doi: 10.1111/wrr.70025 (PMC12008732; doi:10.1111/wrr.70025)

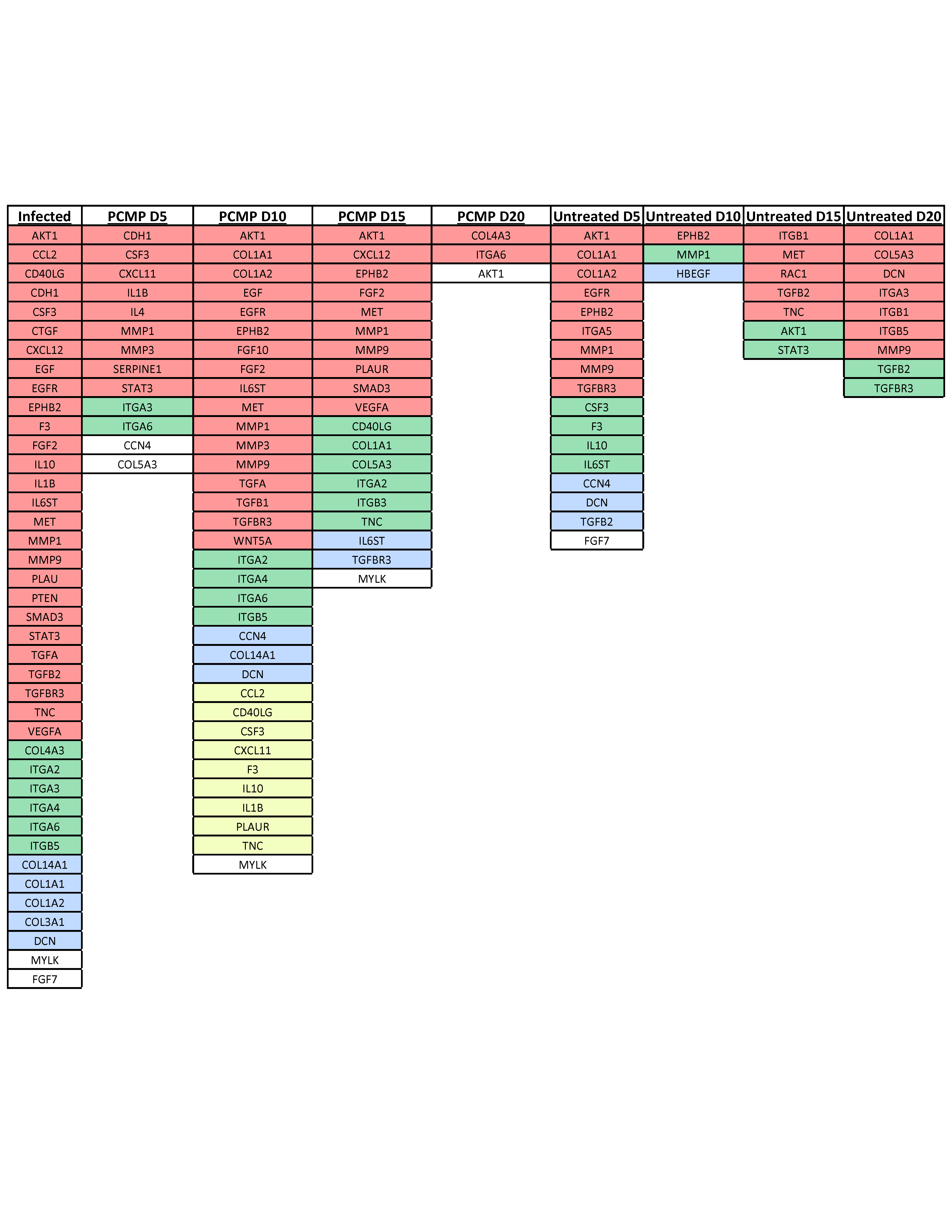

Supplement: Supplementary file 1 — Table S1. STRING assessment genes. Statistically up/down regulated genes were imported into string-db.org and clustered using MCL clustering (inflation parameter 3). [file WRR-33-0-s003.tif]

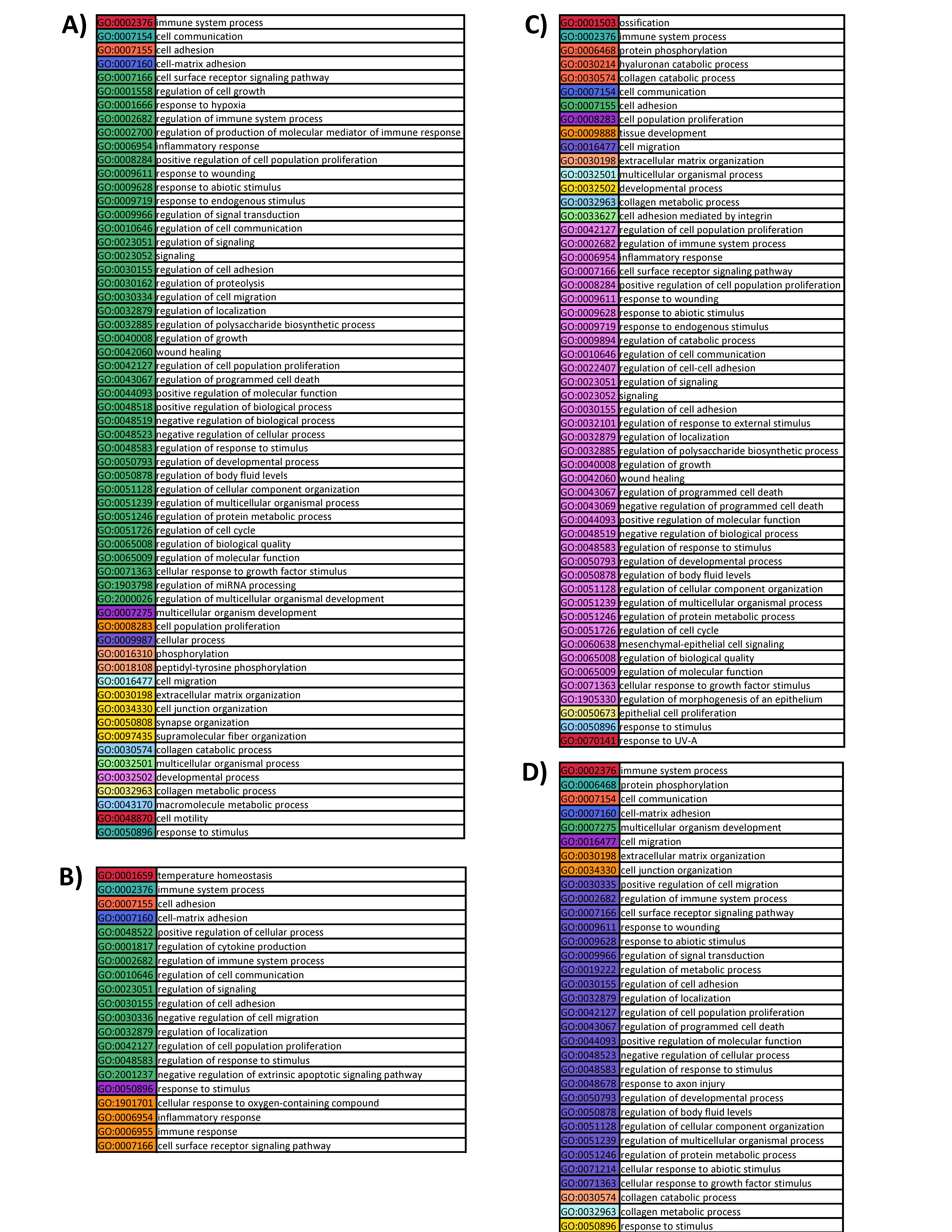

Supplement: Supplementary file 2 — Table S2. REVIGO terms for PCMP wounds. (A) Unwounded versus Infected Day 0; (B) Unwounded versus Day 5 Treated; (C) Unwounded versus Day 10 Treated; (D) Unwounded versus Day 15 Treated. Colour correlates with clustered terms per REVIGO assessment. [file WRR-33-0-s001.tif]

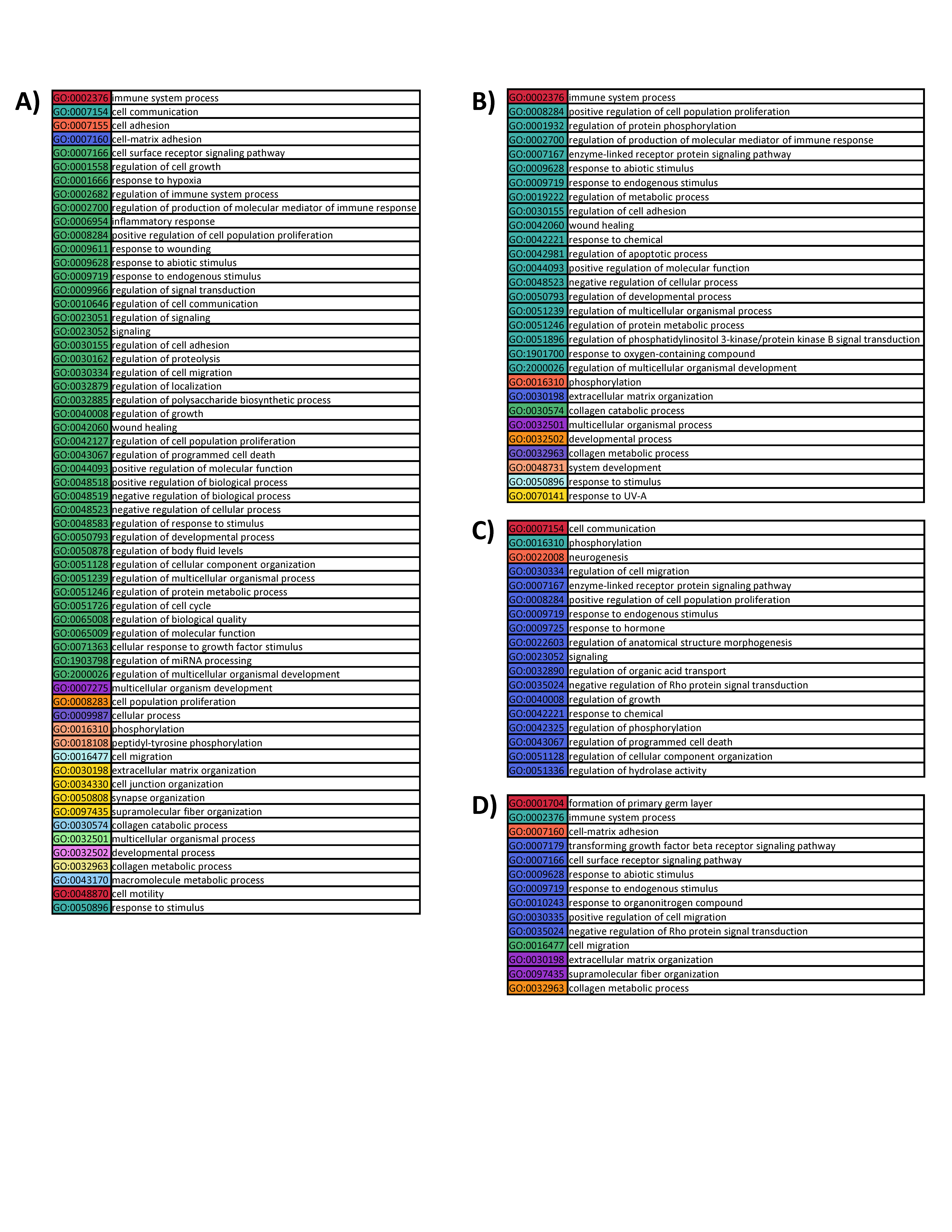

Supplement: Supplementary file 3 — Table S3. REVIGO terms for untreated wounds. (A) Unwounded versus Infected Day 0; (B) Unwounded versus Day 5 Untreated; (C) Unwounded versus Day 15 Untreated; (D) Unwounded versus Day 20 Untreated. Colour correlates with clustered terms per REVIGO assessment. [file WRR-33-0-s002.tif]
